# Pathobiology of HIV-related metabolic and cardiovascular comorbidities: Towards a unifying mechanism

**DOI:** 10.4102/ajlm.v13i1.2582

**Published:** 2024-10-25

**Authors:** Zohreh Jadali

**Affiliations:** 1 Department of Immunology, School of Public Health, Tehran University of Medical Sciences, Tehran, Iran

The recently published *African Journal of Laboratory Medicine* article by Okunorobo et al. brings to light a significant change in the lipid profile of HIV-positive patients versus healthy controls, emphasising the importance of dyslipidaemia in the development of cardiovascular diseases (CVDs).^1^ These results are important, because dyslipidaemia is not only a common metabolic complication for HIV-positive individuals, but it also increases risk for CVDs.^2^ Therefore, understanding the causes of abnormal lipid metabolism is a critical step toward disease management. Unfortunately, there is no definite explanation for why this happens. Comorbidities like dyslipidaemia and CVDs among HIV patients are multifactorial and have no single cause, but insights indicate that immune dysregulation plays a central role in executing pathological conditions.^3^ In essence, there seems to be a connection between dyslipidaemia, CVDs, HIV, and immune-related pathological processes. Among the possible mechanisms, the progressive loss of the CD4+ T-cell population is one of the most important.^4^ Evidence indicates that HIV-positive individuals with low CD4 count have endothelial dysfunction and hypertension that is accompanied by increased CVD risk.^4^ These clinical conditions are strongly influenced by both elements of the immune system and a broad group of lipid components.^4^ HIV-positive individuals also have elevated proportions of T-helper 17 cells, which may promote inflammation, and senescent cells, which may increase risk of cardiovascular disease, in hyperlipidemic conditions.^4^ Interleukin-2 production has also been shown to increase in these patients and can affect lipid metabolism and CVD risk.^4^

Cells of the monocyte-macrophage lineage appear to also play a role in the complicated connection between the immune system and HIV disease, dyslipidaemia, and CVDs.^5^ This is supported by elevation of intermediate/inflammatory monocytes with proatherogenic properties in HIV-positive patients.^5^ These cells play a detrimental role in CVD progression and can express abundant tissue factor, a risk marker for CVDs.^5^ Lipidic environment modification is another reason for production of tissue factor in plaque macrophages; thus, interplay of lipids and immune cells might in part be responsible for the CVD progression in infected patients.^5^ Elevation of macrophage activation markers, soluble CD14, in HIV-positive patients provides additional evidence for the importance of the monocyte-macrophage lineage, because they can be associated with clinical CVD events and dyslipidaemia.^6^

Monocyte and macrophage dysfunction in cholesterol handling as a cause of HIV infection may also affect the CVD risk.^5^ This may occur by down-regulation of adenosine triphosphate-binding cassette transporter A1, which is essential for cholesterol homeostasis and high-density lipoprotein metabolism.^5^

Dysregulated inflammation is another risk factor for both CVD and dyslipidaemia in HIV-positive patients.^7^ This is supported by evidence, including (1) the increased level of high-sensitivity C-reactive protein as a nonspecific inflammatory marker that increases the CVD risk and dyslipidaemia, (2) the increased levels of D-dimer that can indicate a positive association with lipid indicators in patients with coronary artery disease, and (3) the increased production of interleukin-6 that occurs during HIV replication and is associated with dyslipidaemia and increased CVD risk.^7^

Loss of gut barrier function and the ensuing microbial translocation that is usually associated with HIV disease progression can also elicit an inflammatory cascade.^8^ This inflammatory response may induce CVD through alteration in the balance of T-helper 17/regulatory T-cells and their associated cytokines.^9^ T-helper 17/regulatory T-cells appear to be a bridge linking the gut microbiota to host metabolic disorders.^9^

T-helper 17 and regulatory T-cells have antagonistic effects. T-helper 17 cells secrete pro-inflammatory cytokines and promote intestinal inflammation, whereas regulatory T-cells are anti-inflammatory.^10^ Overproduction of pro-inflammatory cytokines could contribute to endothelial damage, and cardiovascular events.^5^ Moreover, lipid profile abnormality due to disturbances in inflammatory and immune processes may exacerbate the process of CVDs in infected individuals.^5^ Microbial translocation is often associated with a reduction in microbial diversity, losing the beneficial microbes, and expansion of the pathogenic microbes.^4^ These changes can result in gut and peripheral T-cells activation and increases in plasma pro-inflammatory factors including tumour necrosis factor-α and soluble CD14.^4^ Increased levels of these molecules confer an increased CVD risk in chronic HIV infection and have a strong link with dyslipidaemia.^4^

Overall, the information above covers only small parts of the whole, but provides evidence for the crucial role of the immune system in dyslipidaemia and CVD risk among HIV patients. Although definite disease mechanisms need to be established, the emergence of unifying mechanisms for HIV and related disorders is not far-fetched, because the immune system is a system of relations that interacts with other body systems. Lipid metabolism and immune system responses regulate each other, and immune-metabolic dysregulation can contribute to HIV-associated pathogenesis and clinical observations related to HIV.^3^ Alternatively, the heart and the immune system are highly integrated systems and immune dysregulation plays an essential role in the development of many CVDs.^3^ Therefore, the harmful effect of HIV infection on an individual’s immune system can disrupt proper functioning of different body systems. However, further investigations are needed to elucidate how the irregularities of the immune system contribute to altered lipid profile and increased risk for CVD among HIV patients.
